# TRPV3 regulates Breast Cancer Cell Proliferation and Apoptosis by EGFR/AKT pathway

**DOI:** 10.7150/jca.93940

**Published:** 2024-03-25

**Authors:** Yan Xie, Hyo In Kim, Qianzhi Yang, Jinghao Wang, Wei Huang

**Affiliations:** 1 Basic Medicine College of Daqing Campus, Harbin Medical University-Daqing, Daqing, 163319, China.; 2 Department of Pharmacology, Hainan Medical University, Haikou, 571199, China.; 3 Department of Surgery, Beth Israel Deaconess Medical Center, Boston, MA, 02215, United States of America.; 4 Department of Pharmacy, The First Affiliated Hospital, Jinan University, Guangzhou, 510630, China.; 5 The Guangzhou Key Laboratory of Basic and Translational Research on Chronic Diseases, Jinan University, Guangzhou, 510630, China.

**Keywords:** breast cancer, TRPV3, EGFR, apoptosis, proliferation

## Abstract

Breast cancer (BC) is one of the most common cancer types worldwide and the first cause of cancer-related deaths in women. Transient receptor potential vanillin 3 (TRPV3) has been preliminarily discovered to play an important role in various cancers, including BC. Here, we explored the effect of TRPV3 on breast cancer cells and its potential mechanism. TRPV3 level was measured in BC tissue and adjacent noncancerous breast tissue using real-time RT-PCR and Western blot. Wound healing was used to detect cell migration. MTT and EDU were detected cell proliferation. TUNEL and Caspase-3 activity were used to detect cell apoptosis. We found that TRPV3 expression significantly increased in both human BC tissues and breast cells line. TRPV3 siRNA (TRPV3 inhibition) dramatically suppressed cell migration and proliferation, promoted the apoptosis, and decreased [Ca^2+^]i; whereas Carvacrol (TRPV3 agonist) has opposite effect in MCF-7 cells. We validated EGFR (Epidermal growth factor receptor) is a direct target protein of TRPV3. Mechanism studies have shown that Carvacrol increased phosphorylation levels of EGFR and AKT, and were decreased by suppression of TRPV3. Moreover, Erlotinib (EGFR inhibitor) and LY294002 (PI3K inhibitor) diminished Carvacrol induced cell migration and proliferation, promoted cell apoptosis, and increased [Ca^2+^]i in Carvacrol group. Our results collectively suggest that TRPV3 siRNA inhibits migration and proliferation, and promoted apoptosis in breast cancer cells by EGFR/AKT pathway. These findings indicate that TRPV3 may represent a novel therapeutic strategy for breast cancer.

## Introduction

Breast cancer (BC), one of the most common carcinoma with a high mortality rate in women worldwide, and its incidence rate has continued to rise in recent years 1. Despite significant progress in the diagnosis and treatment of BC, the 5-year survival rate of BC patients is still very low 2. Therefore, the pathogenesis of BC and new molecular biomarkers need to clarify.

Transient receptor potential (TRP) channels are a class of non-selective cation channel proteins characterized by sharing a common structure of six transmembrane segments and serving as a multimodal cellular sensor for broad-spectrum physical and chemical stimuli 3. Among them, TRPV3 (Transient receptor potential vanilloid 3) is a member of the vanilloid subfamily and has become a new target for cancer treatment interventions 4. TRPV3 inhibits the proliferation and migration of colorectal cancer cells by regulating the MAPK signaling pathway 5. TRPV3 is overexpress in mon-small cell lung cancer and is associated with cancer progression, and promote the angiogenesis of A549 cells by HIF-1α-VEGF signaling pathway 6, 7. Recent reports have shown that TRPV3 is associated with epithelial mesenchymal transition in BC through computer analysis of ion channels 8, but its role in BC is not totally be disclosed.

Epidermal growth factor receptor (EGFR) plays a crucial role in regulating important cellular processes under normal and pathophysiological conditions (such as BC) 9. EGFR overexpression in breast cancer, especially triple-negative breast cancer (TNBC), is associated with large tumor size, poor differentiation, and poor clinical prognosis 10. EGFR is crucial for regulating and maintaining the biological characteristics of cancer, such as proliferation, invasion, and metastasis 11. EGFR inhibitor and monoclonal antibody were treated breast cancer patients 12, 13. Based on the low mutation and high expression of EGFR in cancer, effectively limiting the expression of EGFR protein is conducive to inhibiting the progress of breast cancer 11. Recent reports have shown that TRPV3 enhances the proliferation of skin keratinocytes through the EGFR dependent signaling pathway 14. After treatment with Erlotinib, palmoplantar keratosis in patients with Olmsted syndrome caused by TRPV3 mutations was improved, and TRPV3 signaling leads to EGFR transactivation 15. It is currently unclear whether TRPV3 regulates the progress of breast cancer through EGFR pathway. In this study, we demonstrated that TRPV3 play pivotal role in BC by targeting EGFR, which may provide a potential therapeutic target for the treatment of BC.

## Materials and Methods

### Patients and tissue samples

After approved by the Human Sample Use Ethics Committee of Jinan University, the First Affiliated Hospital of Jinan University provided human breast cancer tissue. These tissues were obtained from 8 patients who underwent surgery for breast tumors and adjacent non tumor tissues. The sampled tissue was immediately frozen in liquid nitrogen and stored at -80°C.

### Cell culture

Human breast cancer cell (MCF-7, MD-MB-231, MDA-MB-468 and SK-BR3) and non-neoplastic epithelial cell lines (MCF-10A) purchased from the cell bank of the Chinese Academy of Sciences (Shanghai, China). The cells cultured in Dulbecco Modified Eagle Medium (DMEM) containing 10% fetal bovine serum (FBS, Hyclone, USA) at 37 °C and 5% CO2.

### siRNA transfection

During the transfection process, MCF-7 cells were starved in serum-free DMEM for 24 hours, and then transiently transfected with TRPV3 siRNA (Santa Cruz Biotechnology, USA) and negative control (NC, non-sensitive interference sequence of TRPV3) at a concentration of 100 nM (Libo Biological Co., Ltd., Guangzhou, Guangdong Province, China). The X-treme RNA transfection reagent (Invitrogen, USA) is used as a transfection vector according to the manufacturer's agreement. Cells harvested 48 hours after transfection. Carvacrol was purchased from Sigma (St. Louis, Missouri, USA), LY294002 from Beyotine (Shanghai, China), and Erlotinib from Med Chem Express (Shanghai, China).

### Cell viability assay

Cells (2 × 10^4^ cells/well) inoculated into a 96-well culture plate. According to the manufacturer's instructions, cell viability was measured using the 3-(4,5-dimethylthiazol-2-yl)-2,5-diphenyltetrazolium bromide (MTT) assay. Measure absorbance at 490nm. Cell viability is expressed as the relative living cells relative to the control cells (%).

### Cell migration

For wound healing analysis, cells inoculated into a 6-well plate 24 hours after transfection. After 24 hours, use the tip of the plastic pipette (10 μl) damages the adhesive cells. Then rinse the excretory cells with PBS and culture them in serum-free DMEM for 24 hours. Take photos of wound closure in different groups and evaluate using a microscope.

### Terminal deoxynucleotidyl transferase dUTP nick end labeling (TUNEL)

According to the manufacturer's instructions, apoptosis of MCF-7 detected using the in situ cell death detection kit (TUNEL fluorescence FITC kit, Roche, USA). After TUNEL staining, MCF-7 cells stained using DAPI (Sigma Aldrich) and observed using laser scanning confocal microscopy (Olympus, Fluoview1000, Tokyo, Japan). The number of apoptotic cells expressed as a percentage of the total cells counted.

### Quantitative real-time PCR (qRT-PCR)

TRPV3 mRNA level was measured by qRT-PCR. Extract total RNA from cells using the Trizol (Invitrogen, Carlsbad, USA) method. According to the manufacturer's instruction, the SYBR Green Master Mix Kit (Applied Biosystems, USA) used for relative quantification of RNA levels. GAPDH selected as internal control. TRPV3 forward primer, 5'-CGGATCCGATGGTGTGTGTGGGAGTCCCCG-3'; TRPV3 reverse primer, 5'-CCTCCGAGGCTAAAGTTTGGCCTTGA-3'. GAPDH forward primer, 5'-AAGAGGTGTGGAAGGC-3'; GAPDH reverse primer, 5'- TCACCACCCAGTTGCTGTA-3'.

### EdU fluorescence staining

5-ethynyl-2-neneneba deoxyuridine (EdU) fluorescence staining used to detect the newly synthesized DNA in MCF-7 cells after indicator treatment. All steps carried out according to the manufacturer's instructions for the Cell Light EdU DNA Cell Proliferation Kit (RiboBio, Guangzhou, China).

### Immunofluorescence staining

MCF-7 cells washed with PBS, then fixed with 4% PFA for 20 minutes, and transduced with 0.1% TritionX-100 at room temperature (20 minutes). Then, they treated with 5% bovine serum albumin (BSA) to block non-specific binding and incubated overnight in the desired antibody at 4 °C. Incubate the cells in a fluorescent label second antibody for 60 minutes. DAPI (Sigma Aldrich, USA) used to label the nucleus. Then, the slide analyzed using a laser scanning confocal microscope (Olympus, Japan).

### Western blot analysis

Extract total protein samples from MCF-7 cells and cancer tissue for Western blot analysis. The protein sample (50 µg) graded by SDS-PAGE (10% polyacrylamide gel) and transferred to the nitrocellulose membrane. Seal the membrane in 5% skim milk PBS for 2 hours, and then incubate overnight with the following primary antibodies: TRPV3 (1:100, Abcam, USA), as well as p-EGFR, EGFR, p-AKT, and AKT (1:500, Cell Signaling Technology, USA) and GAPDH (1:1000, ZSJZ Bio, Beijing, China). After washing, incubate the membrane with the second antibody for 1 hour. The image captured on the Odyssey CLx infrared imaging system (LI-COR Biosciences, Lincoln, NE, USA). Quantitative analysis of protein imprinting bands using Odyssey CLx v2.1 software. Standardize data into GAPDH as an internal control.

### Measurement of [Ca^2+^]i

Cells were incubated with 10 µM Fluo-3/AM (acetoxymethyl ester, Molecular Probes, China) working solution containing 0.03% Pluronic F-127 at 37°C for 40 minutes. Subsequently, wash the cells with working solution to eliminate extracellular Fluo-3/AM. Determine the changes in [Ca^2+^]i by fluorescence intensity (FI). The FI of these cells detected using a laser scanning confocal microscope (Olympus, Japan), excited at 488nm, and emitted at 530nm. Collect FI from 10 randomly selected cells to calculate the average FI.

### Co-immunoprecipitation

The cells were lysed in lysis buffer (Aspen Biological) and approximately 180 μg of total cellular proteins were incubated overnight with target antibody at 4 °C, then added 20 μl protein A agarose beads (BIO-RAD, USA). Rabbit control IgG (Abclonal, China) used in the reaction as the negative control. After centrifugation, collect the beads and gently rinse them. Denature the sample and analyze it using SDS-PAGE to detect the interaction between two protein partners.

### Caspase-3 activity assay

According to the manufacturer's instructions, the activity of caspase-3 in MCF-7 cells measured using a colorimetric assay kit (Beyotime Institute of Biotechnology, China). MCF-7 cells were lysed in ice-cold cell lysis buffer for 15min, and then centrifuged at 20,000×g for 10min at 4°C. Add 30μl supernatant and 10 μl substrate (2 mM Ac-DEVD-pNA) at 60 μl buffer at 37°C for 2h. The absorbance measured at 405nm.

### Data analysis

Group data expressed as mean ± SEM. Using unpaired Student's t-test or one-way ANOVA to determine statistical significance, post hoc Dunnett's comparison only performed when the P-value of F in the ANOVA is less than 0.05 and there is significant variance heterogeneity. Differences were considered to be statistically significant when P < 0.05.

## Results

### TRPV3 significantly increased in human breast cancer tissues and cell lines

**Figure 1:**
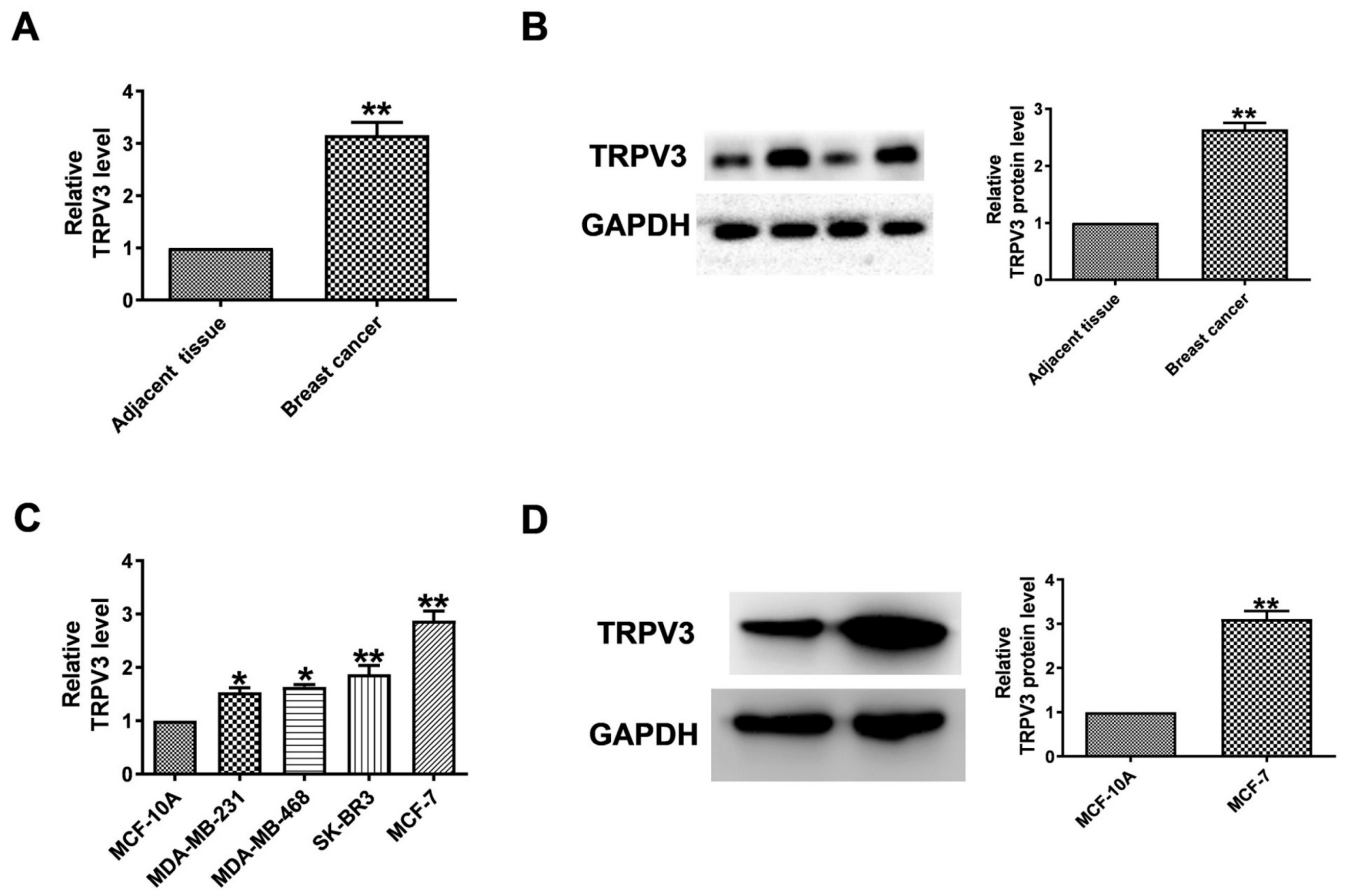
TRPV3 is upregulated in breast cancer. (A) Expression levels of TRPV3 mRNA. n=8. ^**^P < 0.01 vs. adjacent breast tissue. (B) TRPV3 protein expression in breast cancer and adjacent breast tissue. n=6. ^**^P < 0.01 vs. adjacent breast tissue. (C) TRPV3 mRNA levels in MDA-MB-231, MDA-MB-468, SK-BR3, MCF-7 and MCF-10A cell lines. n=4. ^*^p< 0.05, ^**^p< 0.01 vs. MCF-10A. (D) TRPV3 protein expression in MCF-7 cells. n=3. ^**^p< 0.01 vs. MCF-10A.

To evaluate the level of TRPV3 in breast cancer, qRT-PCR and Western blot to quantify its expression in breast cancer tissues and normal adjacent tissues. TRPV3 was significantly increased in breast cancer specimens (Fig. 1A, B). Then, we tested the expression level of TRPV3 in several breast cancer cell lines (MCF-7, SK-BR3, MDA-MB-468, MDA-MB-231). Compared with human mammary epithelial cells (MCF-10A), MCF-7 cells showed the most significant upregulation of TRPV3 (Fig. 1C). The level of TRPV3 protein in MCF-7 cells was also significantly increased with increasing mRNA levels subsequently (Fig. 1D). The results showed that the overexpression of TRPV3 might play an important role in the process of breast cancer delivery.

### TRPV3 siRNA inhibits cell proliferation and migration, and promotes cell apoptosis

**Figure 2:**
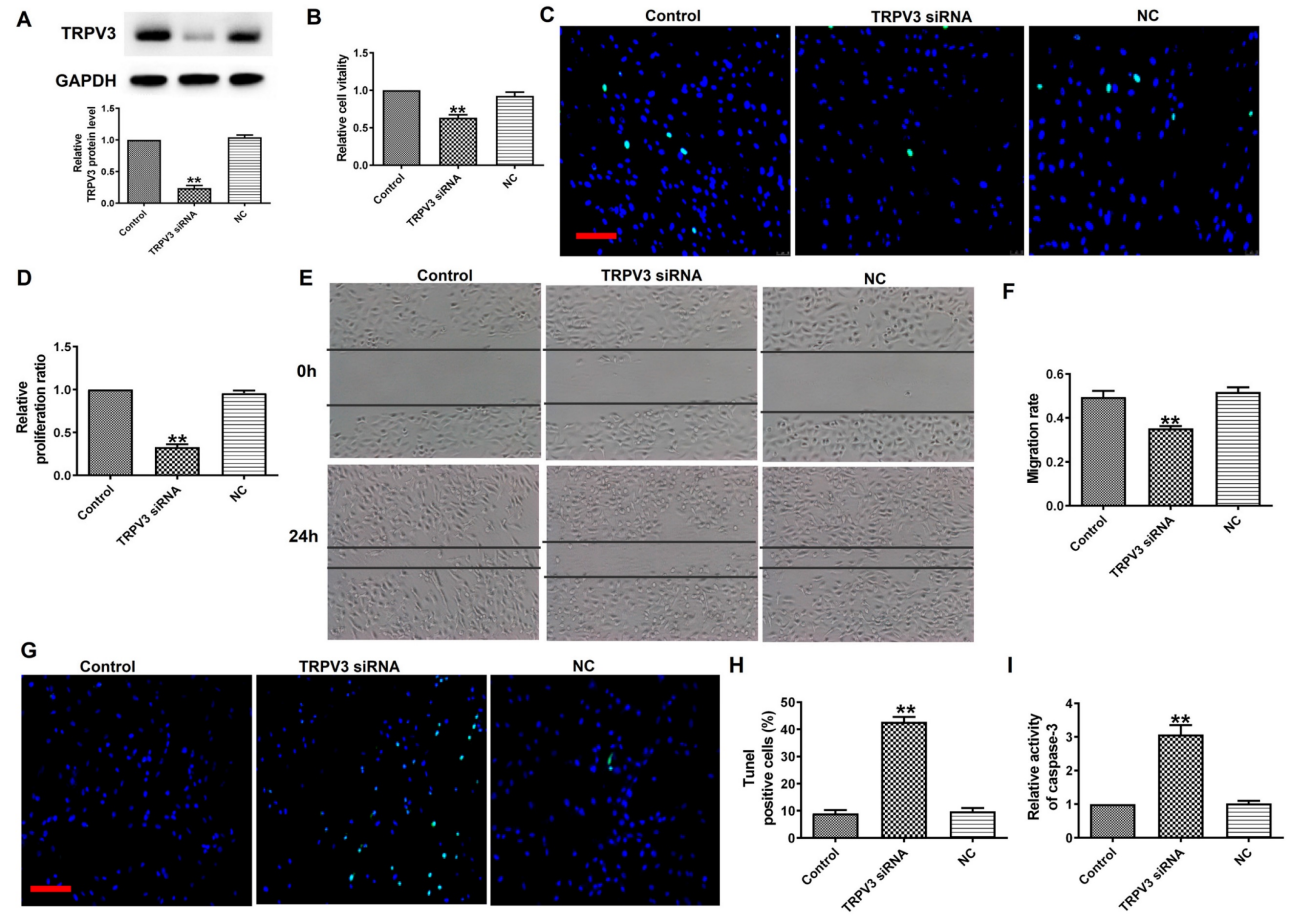
TRPV3 siRNA inhibits MCF-7 cell viability, migration and proliferation, and promotes cell apoptosis. (A) TRPV3 protein expression after TRPV3 siRNA transfection in MCF-7 cells. n=3. (B) MTT assay. n=4. (C, D) Proliferation of breast cancer cells after TRPV3 siRNA transfection for 48 h by EDU assay. Scale bar=100μm. n=4. (E, F) Migration of breast cancer cells after TRPV3 siRNA transfection for 24 h by Wound healing assay. n=4. (G, H) Apoptosis of breast cancer cells after TRPV3 siRNA transfection for 48 h by TUNEL staining. Apoptotic cells are green, and the nuclei are stained blue with DAPI. Scale bar=100 μm. n=4. (i) Caspase-3 activity. n=4. ^**^P < 0.01 vs MCF-7 and miRNA NC groups.

To explore the functional role of TRPV3 in BC, we transfected TRPV3 siRNA into MCF-7 cells to induce TRPV3 inhibition (Fig. 2A). Next, MTT analysis and EDU assay demonstrated that TRPV3 siRNA remarkably inhibited the activity of MCF-7 cells (Fig. 2B) and blocked MCF-7 cell proliferation (Fig. 2C, D). In addition, wound healing showed that TRPV3 siRNA experienced slower wound closure compared to the NC or control group (Fig. 2E, F). Moreover, the TUNEL revealed that the number of apoptotic positive cells was increased in cultured breast cancer cells transfected with TRPV3 siRNA (Fig. 2G, H). Finally, we measured the changes of caspase-3 activity in MCF-7 cells after TRPV3 siRNA transfection. Caspase-3 is a key regulator of apoptosis in the process of breast cancer 16. As shown in Fig. 2I, TRPV3 siRNA increased the level of caspase-3 activity in MCF-7 cells. In conclusion, these results suggest that TRPV3 siRNA may play a role as a tumor inhibitory tool by inhibiting cell migration and proliferation, and promoting apoptosis in breast cancer cells.

### TRPV3 interacts with the EGFR and modulates EGFR/AKT activation

**Figure 3:**
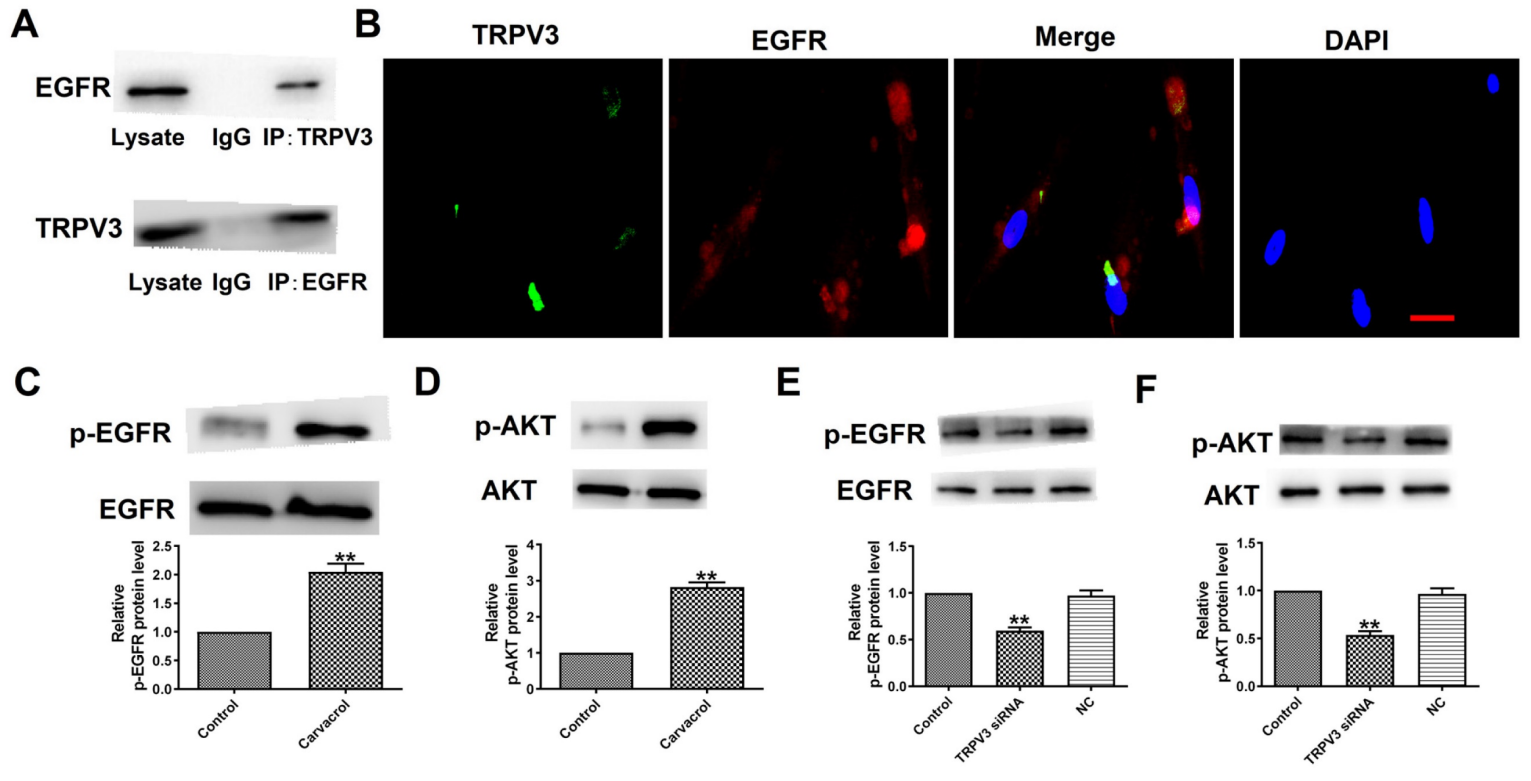
TRPV3 interacts with the EGFR and modulates EGFR/AKT activation. (A) Interaction of TRPV3 with EGFR in breast cells analyzed by co-IP. IgG, immunoglobulin G. n=3. (B) Representative immune-fluorescent staining of MCF-7cells with specific antibodies against TRPV3 (green), EGFR (red), and DAPI (blue) to label nuclei. Scale bar=50nm. n=4. (C, D, E, F) Phosphorylation levels of EGFR and AKT levels determined by Western blot. n=3. ^**^P < 0.01 vs. Control groups.

To explore the downstream cell signals of TRPV3, we first conducted bioinformatics analysis using a string database (https://cn.string-db.org) to identify TRPV3 containing potential binding sites for EGFR. Our co-IP experiment confirmed that anti TRPV3 or anti EGFR antibodies can reduce each other's chaperone proteins (Fig. 3A). Consistent with our co-IP results, TRPV3 and EGFR were co-localize in cultured MCF-7 cells under confocal microscopy (Fig. 3B). The overexpression of EGFR in cancer positively correlated with pathological progression, which is an important marker of BC 10. One of the main signaling pathways of EGFR activation is the activation of AKT serine-threonine kinases, which leads to the induction of proliferation, growth and inhibition of apoptosis in breast cancer 16. Further proving whether EGFR/AKT is a downstream regulatory target of TRPV3, we used Western blot to monitor the expression levels of p-EGFR and p-AKT in MCF-7 cells. The results demonstrated that Carvacrol treatment significantly increased the levels of p-EGFR and p-AKT in MCF-7 cells (Fig. 3C, D) which were suppressed by TRPV3 siRNA transfection, but not by disrupted siRNA NC inhibition (Fig. 3E, F), suggesting that TRPV3 induces EGFR/AKT activation in MCF-7 cells.

### TRPV3 regulates cell migration, proliferation, and apoptosis through EGFR/AKT signaling pathway

**Figure 4:**
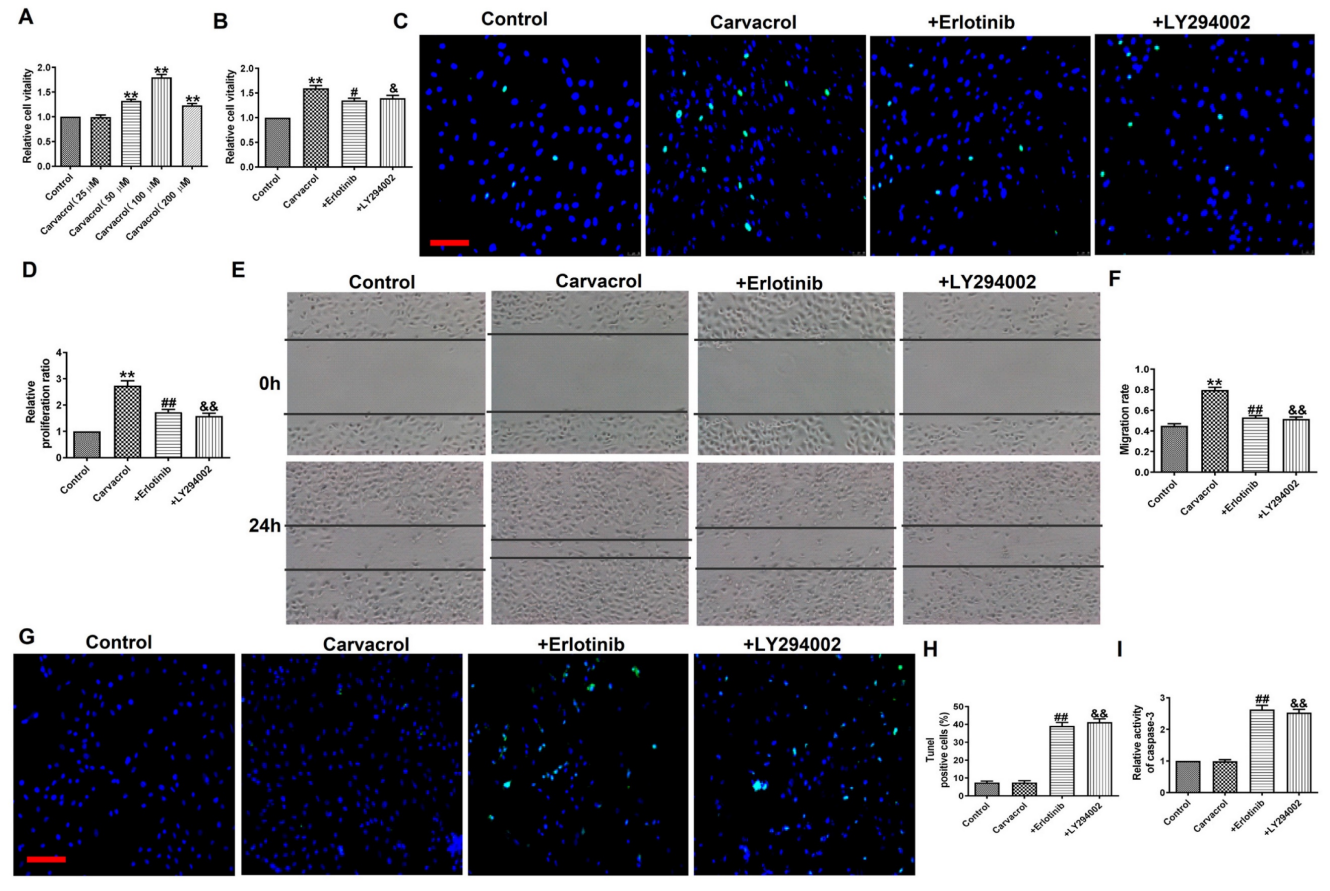
TRPV3 regulated cell migration, proliferation, and apoptosis through EGFR/AKT signaling pathway. (A, B) MTT assay. (C, D) Migration of breast cancer cells by Wound healing assay. (E, F) Proliferation of breast cancer cells by EDU assay. Scale bar=100 μm. (G, H) Apoptosis of breast cancer cells by TUNEL staining. Apoptotic cells are green, and the nuclei are stained blue with DAPI. Scale bar=100μm. (I) Caspase-3 activity. ^**^P < 0.01 vs. Control; **^#^**P < 0.05, **^##^**P < 0.01, ^&^P < 0.05, ^&&^P < 0.01 vs. Carvacrol. n = 4.

To determine whether EGFR/AKT mediates the response of cells to TRPV3 activation, we added EGFR or AKT inhibitors to the cells based on the addition of Carvacrol to validate them in MCF-7 cells. MTT analysis (Fig. 4A) shows that compared to the control group, 25 and 100 μM of Carvacrol causes a concentration dependent increase in cell viability. However, at the higher concentration of 200 μM, Carvacrol seems to reduce cell viability compared to 100 μM. In the following experiment, we selected 100 μM Carvacrol as the final concentration. Carvacrol increased cell viability which were inhibited by EGFR inhibitor, Erlotinib (100 nM) and PI3K inhibitor, LY294002 (3 μM) in MCF-7 cells (Fig. 4B). We further detected cell proliferation and migration through EDU analysis and wound healing (Fig. 4C-F). The results showed that Carvacrol had a serious promoting effect on the proliferation and migration of MCF-7 cells, while elotinib and LY294002 retained this promoting effect (Fig. 4C-F). Subsequently, treatment with Erlotinib and LY294002 promoted apoptosis and significantly increased the activity of caspase-3 in Carvacrol induced MCF-7 cells (Fig. 4G-I). In conclusion, these results indicate that TRPV3 mediates cell migration, proliferation and apoptosis by targeting EGFR/AKT.

### TRPV3 regulates [Ca^2+^]i by EGFR/AKT signaling pathway

**Figure 5:**
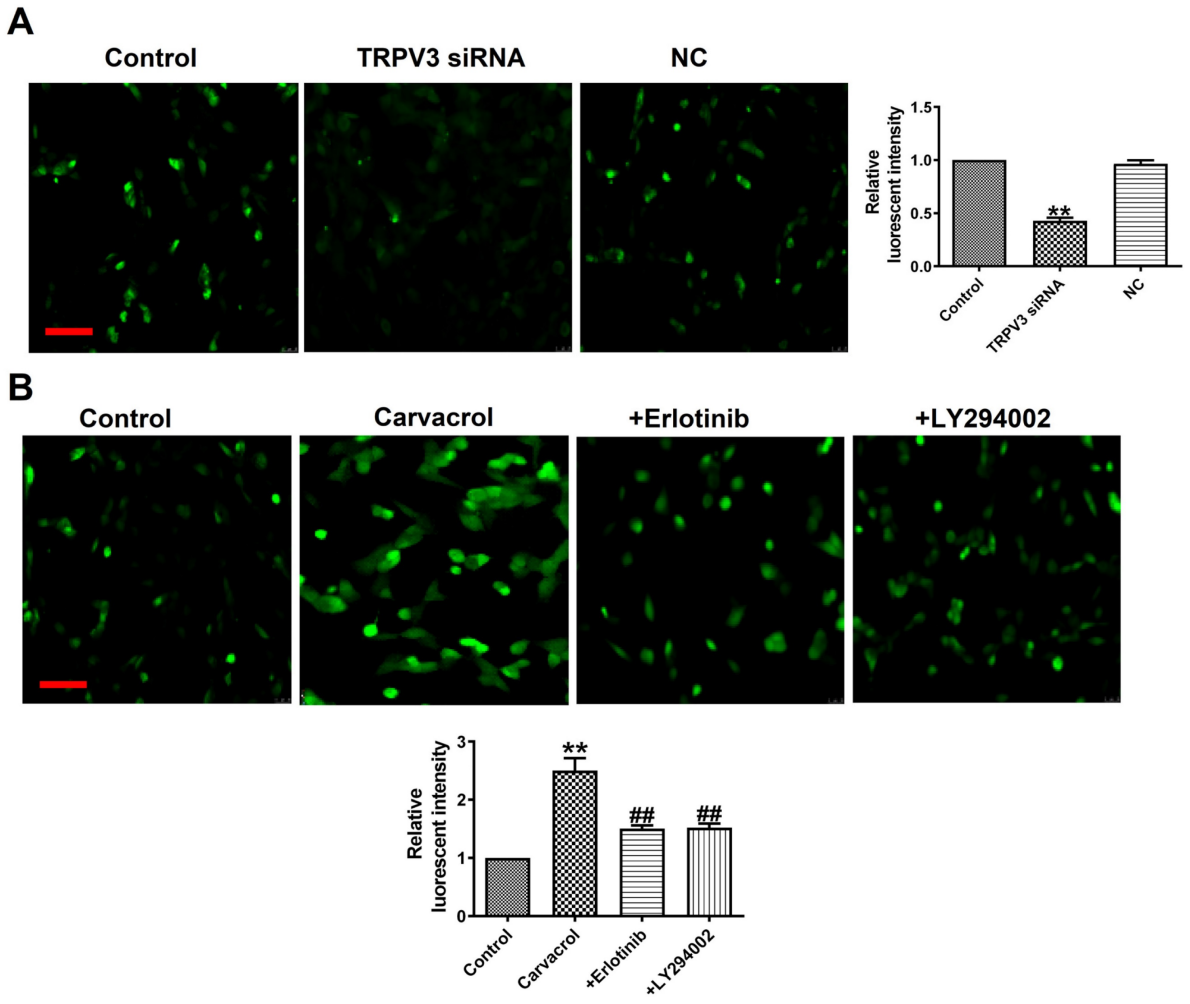
TRPV3 regulates [Ca^2+^]i by EGFR/AKT signaling pathway. (A, B) Fluorescent intensity in [Ca^2+^]i was recorded by laser scanning confocal microscope. Scale bar=100μm. n=4.^ **^P < 0.01 vs. Control; **^##^**P < 0.01 vs. Carvacrol.

What is the mechanism by which TRPV3 regulates cell migration, proliferation, and apoptosis through the EGFR/AKT pathway? Ca^2+^ influx plays an important role in tumorigenesis and the maintenance of a variety of cancer hallmarks, including cell migration, proliferation, invasion or apoptosis in breast cancer 17. The increase in cytoplasmic Ca^2+^ concentration ([Ca^2+^]i) is necessary for activating multiple physiological functions, such as proliferation, exocytosis, and gene transcription 17. From fluorescence intensity taken by laser scanning confocal microscope, we found that Carvacrol treatment significantly increased [Ca^2+^]i, and decreased [Ca^2+^]i through TRPV3 siRNA in breast cancer cells (Fig. 5A), which suggesting that the effect of TRPV3 in MCF-7 cells is significantly associated with [Ca^2+^]i. To determine whether EGFR/AKT mediates [Ca^2+^]i in response to TRPV3, we found Erlotinib or LY294002 reversed the increase of [Ca^2+^]i after using of TRPV3 agonist Carvacrol (Fig. 5B). In conclusion, these results indicate that TRPV3 mediates cell [Ca^2+^]i by EGFR/AKT signaling pathway.

## Discussion

In this study, we found that TRPV3 significantly up-regulation in breast cancer and MCF-7 cells. TRPV3 siRNA significantly inhibits the migration and proliferation, promoting cell apoptosis of MCF-7 cells. In addition, we also revealed a new regulatory mechanism between TRPV3 and the EGFR/AKT pathway. These findings suggest that TRPV3 inhibition may exert a cancer suppressive effect by directly targeting EGFR in cancer.

In breast cancer, the expression and functional changes of TRP channels directly related to cellular processes that promote cancer progression, such as cell proliferation, differentiation, angiogenesis, migration, invasion, and apoptosis induced cell death 18. Some studies have shown that agonists and antagonists can regulate the activity of TRP channels in breast cancer with or without chemotherapy drugs, which indicates that they are good therapeutic targets 18-22. TRPM7 regulates the migration and invasion of metastatic breast cancer cells through the MAPK pathway 19. Overexpression of TRPC1 inhibits proliferation and migration of ER+ breast cancer, and provides better prognosis by inhibiting the activation of PI3K/AKT pathway 20. The activation of TRPV1 significantly inhibits the growth of breast cancer cells and induces apoptosis and necrosis 21. TRPV4 may regulate breast cancer metastasis by regulating cell flexibility and extracellular protein expression through calcium dependent AKT-E-cadherin signal axis 22. Although TRPV3 is a member of TRP superfamily, through network analysis, TRPV3 related to epithelial mesenchymal transformation of breast cancer 8, but the role and mechanism of TRPV3 in breast cancer have not fully clarified. We observed that TRPV3 upregulated in human breast cancer samples and cell lines. TRPV3 inhibition (TRPV3 siRNA) exert its pro-apoptotic activity (the number of apoptotic positive cells and caspase-3 activity were remarkably increased), anti-migration and anti-proliferation, and cell viability decreased in breast cancer cells. However, the number of apoptotic cells and caspase-3 activity were remarkably increased, and cell migration and proliferation rate inhibited as Carvacrol added to cells.

In order to determine the mechanism of TRPV3 mediated migration, proliferation and apoptosis of breast cancer cells, we identified EGFR as a key mediator. We first conducted bioinformatics analysis, and a study showed that EGFR signaling regulated by TRPV3 through direct protein-protein interactions 23. We identified EGFR as a direct target of TRPV3 using co-IP and co-localization analysis. EGFR overexpression can regulate several downstream cancer promoting signaling pathways, including the PI3K/AKT pathway 24. The EGFR/AKT pathway is a key pathway closely related to proliferation, invasion, and angiogenesis, ultimately leading to the malignant biological behavior of BC 25, 26. Downregulation of p-EGFR and p-AKT protein levels observed in cells transfected with TRPV3 siRNA. However, upregulation of p-EGFR and p-AKT protein levels observed in Carvacrol treated cells. In addition, Erlotinib or LY294002 could reversed the anti-apoptotic, and pro-migration and pro-proliferation effects of Carvacrol in MCF-7 cells. These results show that TRPV3 regulates cell migration, proliferation and apoptosis by EGFR/AKT signaling pathway.

In tumor diseases, including BC, intracellular Ca^2+^ dependent signaling is involved in various processes that promote tumor occurrence and progression, such as proliferation, migration, angiogenesis, and apoptosis 4. Some studies have shown that TRP channels related to the regulation of intracellular Ca^2+^ concentration play a role in the progression of breast cancer 4, 27-29. TRPC1 silencing leads to reduction of Ca^2+^ signal mediated breast cancer process induced by FGFR1 activation 27. TRPC6 interacts with and participates in STIM2 to maintain cytosolic and endoplasmic reticulum Ca^2+^ concentrations in MCF-7 cells, thus mediating apoptosis 28. TRPV6 overexpression increases the basal intracellular levels of Ca^2+^ in breast cancer cells, and positively regulates the expression of regulator gene through CaM/Calcineurin/NFAT pathway, which regulates proliferation and apoptosis 29. In this study, we studied the intracellular levels of Ca^2+^ by Carvacrol or TRPV3 siRNA in breast cancer cells. Carvacrol stimulated the increase of intracellular Ca^2+^concentration, but was inactive in stimulating Ca^2+^ by TRPV3 siRNA in breast cancer cells, demonstrating the opposite effect of Carvacrol and TRPV3 siRNA in BC progression (migration, proliferation and apoptosis). In addition, Erlotinib or LY294002 could reversed the increase of Ca^2+^ in Carvacrol group in MCF-7 cells. These results show that TRPV3 regulates cell [Ca^2+^]i by EGFR/AKT pathway.

To sum up, our results show that TRPV3 up-regulated in breast cancer, and inhibiting TRPV3 significantly promotes MCF-7 cell apoptosis, inhibits cell migration and proliferation through targeting EGFR/AKT pathway. However, it is undeniable that, in addition to our findings, the effect of TRPV3 on cancer also mediated by multiple molecular signaling pathways. In addition, the relative importance of TRPV3 directly targeting other proteins or transcription factors in the development of BC is currently unclear. Next, we will conduct experiments on animals to continue to observe the effect of TRPV3 on breast cancer. Collect animal samples for transcriptomics or proteomics detection, and further study the deeper mechanism of TRPV3 regulating breast cancer through experiments. In summary, this study reveals a new mechanism of the anti-tumor effect of TRPV3 siRNA, which may be a potential therapeutic drug for BC treatment.
